# Fifteen Years’ Use of Patient-Reported Outcome Measures at the Group and Patient Levels: Trend Analysis

**DOI:** 10.2196/15856

**Published:** 2019-09-30

**Authors:** Niels Henrik I Hjollund

**Affiliations:** 1 AmbuFlex/WestChronic, Occupational Medicine University Research Clinic Aarhus University Herning Denmark; 2 Department of Clinical Epidemiology Aarhus University Hospital Aarhus Denmark

**Keywords:** patient-reported outcome, questionnaires, chronic disease, outpatient follow-up, patient involvement, resource reallocation

## Abstract

**Background:**

Since 2004, we have collected patient-reported outcome (PRO) data from several Danish patient populations for use at the group and patient levels.

**Objective:**

The aim of this paper is to highlight trends during the last 15 years with respect to patient inclusion, the methods for collection of PRO data, the processing of the data, and the actual applications and use of the PRO measurements.

**Methods:**

All PRO data have been collected using the AmbuFlex/WestChronic PRO system, which was developed by the author in 2004 and has been continuously updated since. The analysis of trends was based on a generic model applicable for any kind of clinical health data, according to which any application of clinical data may be divided into four processes: patient identification, data collection, data aggregation, and the actual data use. Data for analysis were generated by a specific application in the system and transferred for analysis to the R package.

**Results:**

During the 15-year period, 78,980 patients within 28 different groups of chronic and malignant illnesses have answered 260,433 questionnaires containing a total of 13,538,760 responses. Several marked changes have taken place: (1) the creation of cohorts for clinical epidemiological research purposes has shifted towards cohorts defined by clinical use of PRO data at the patient level; (2) the development of AmbuFlex, where PRO data are used as the entire basis for outpatient follow-up instead of fixed appointments, has undergone exponential growth and the system is currently in use in 47 International Statistical Classification of Diseases and Related Health Problems groups, covering 16,000 patients and 94 departments throughout Denmark; (3) response rates (up to 92%) and low attrition rates have been reached in group level projects, and there are even higher response rates in AmbuFlex where the patients are individually referred; (4) The answering method has shifted, as while in 2005 a total of 66.5% of questionnaires were paper based, this is the case for only 4.3% in 2019; and (5) the approach methods for questionnaires and reminders have changed dramatically from letter, emails, and short message service text messaging to a national, secure electronic mail system through which 93.2% of the communication to patients took place in 2019. The combination of secure email and web-based answering has resulted in a low turnaround time in which half of responses are now received within 5 days.

**Conclusions:**

The demand for clinical use of PRO measurements has increased, driven by a wish among patients as well as clinicians to use PRO to promote better symptom assessment, more patient-centered care, and more efficient use of resources. Important technological changes have occurred, creating new opportunities, and making PRO collection and use cheaper and more feasible. Several legal changes may constitute a barrier for further development as well as a barrier for better utilization of patients’ questionnaire data. The current legal restrictions on the joint use of health data imposed by the European Union’s General Data Protection Regulation makes no distinction between use and misuse, and steps should be taken to alleviate these restrictions on the joint use of PRO data.

## Introduction

From the time of Hippocrates, information originating from the patient has been considered indispensable. Today, few diagnoses can be established, and few treatments monitored sufficiently, solely by using paraclinical data without explicit information from the patient. However, until recently, such patient inputs were always shortened and interpreted by a clinician. With the introduction of the term health-related quality of life, systematic measurement was adopted for research in several clinical specialties [1]. The potential of its many applications was further boosted by the US Food and Drug Administration’s definition of a patient-reported outcome (PRO) as a measurement based on:

Any report of the status of a patient’s health condition that comes directly from the patient, without interpretation of the patient’s response by a clinician or anyone else [2].

The first draft of this term appeared in 2006 [3] and the final version in 2009 [2]. Since 2004, we have collected PRO data (although the term PRO data was not coined at that time) from several Danish patient populations. The aim of this paper is to highlight trends during the last 15 years with respect to patient inclusion, the collection of PRO data, the processing of the data, and the actual applications and use of the PRO measurements.

## Methods

### Overview

All PRO data have been collected using the AmbuFlex/WestChronic PRO system, which was developed by the author in 2004 and has been continuously updated since. The system has been described in detail elsewhere [4,5]. The first version of the generic PRO system, WestChronic, was developed for mixed-mode (Web and paper) collection of PRO data for research purposes in clinical epidemiological studies with repetitive measurements. Based on experienced feasibility and high response rates, it was decided in 2007 to develop this system into a flexible, multipurpose PRO system. The goal was to use clinical PRO data as the basis for outpatient follow-up in selected patient groups.

### AmbuFlex: Telehealth Patient-Reported Outcomes as the Basis for Follow-Up in Chronic Diseases

In AmbuFlex, outpatients report their symptoms from home at regular intervals instead of attending fixed visits at the outpatient clinic. The PRO measures are used to decide whether a patient needs or wishes an outpatient visit, and were developed to promote better symptom monitoring, more patient-centered care, and more efficient use of resources [5]. Specific questionnaires have been developed for each diagnostic group. The AmbuFlex concept consists of three generic elements: PRO data collection, PRO-based automated decision algorithm, and PRO-based graphical overview for clinical decision support.

### The AmbuFlex/WestChronic Patient-Reported Outcomes System

The AmbuFlex/WestChronic system supports dynamic mixed-mode data collection with the use of the internet or paper forms, as well as automated communication to the patient and the clinician via personalized letters, emails, text messages, and secure electronic communication. All information regarding implemented projects, items and questionnaires, communication, clinical users, and patients resides in tables in a Structured Query Language database residing in the server park of Region Central Denmark. All administration of projects, questionnaires, users, and patients is supported by the system’s software and managed in browser windows.

**Figure 1:**
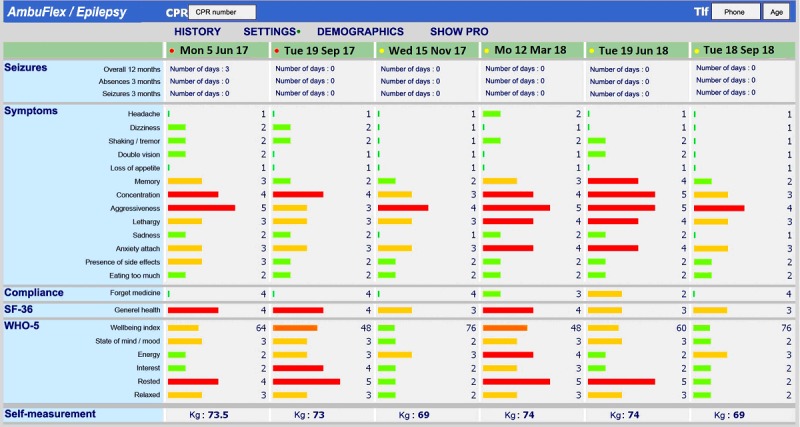
Example of the symptom overview in AmbuFlex/Epilepsy. The bar color and length both indicate the severity of the symptom (translated from Danish).

The system has several integrations (Table 1 and Figure 1). All Danish citizens are assigned a unique 10-digit number (Civil Personal Registration [CPR] number), and continuously updated information on their current postal address and vital status is available from the Danish Civil Registration System [6]. This information is automatically collected online prior to any approach to patients. On-demand printing of questionnaires and letters, as well as scanning of incoming questionnaires with subsequent optical character recognition, is controlled by the system software, and results about all variables end up in result tables for the individual implemented projects in the same database. This occurs irrespective of whether Web or paper forms are used, and all results are instantaneously accessible. WestChronic may implement an arbitrary number of PRO projects with individual protocols, questionnaires, patients, and clinical users. For the patient and clinician, each implemented project appears as a unique PRO project with its own logo, domain, website, accompanying letters, contact information, etc. A new and rewritten version is underway.

**Table 1:** The AmbuFlex/WestChronic PRO system’s online integrations with other systems.

System	Purpose
Electronic Health Record system^a^	Clinicians may access a graphical overview of the patient’s PRO^b^ measurements in AmbuFlex with a single click from the patient’s record in the EHR^c^ (Figure 1).
Danish Civil Registration System	Information on current address and vital status, including possible date of death.
The national health portal (sundhed.dk)	Patients may, after secure login at the portal, access the same PRO overview and data as the clinician (Figure 1).
Email	Automated emailing of reminders, etc (obsolete).
Text messaging	Reminders and secure login (two-factor authentication).
Secure electronic mail (e-boks)	Automated mailing of links to questionnaires and reminders.
Paper questionnaire printing and scanning	Automated printing of individualized letters and questionnaires. Automated optical character recognition of received questionnaires.
Health data network (SDN)	A national secure virtual private network connecting hospitals, health data providers, etc.
Single-sign-on	Enables clinicians in other regions to login using their usual credentials.

^a^Available in three of the five Danish Regions. In the other two regions, AmbuFlex appears as a separate system.

^b^PRO: patient-reported outcome.

^c^EHR: electronic health record.

### Data Analysis

The data for the present paper were generated by an *ad hoc* application in the system and transferred for analysis in the R package (R Foundation for Statistical Computing, Vienna, Austria). Patients who had been referred but not yet answered at least one questionnaire were not included. The data collection in the first project started in September 2004, and during 2004 a total of 301 questionnaires were collected from 241 patients; this period was considered a pilot phase and not included in the analysis.

**Figure 2:**
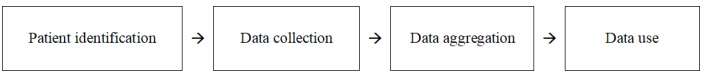
The four processes in the lifespan of patient-related health data (from [7]).

The analysis of trends was based on a generic model applicable for any clinical health data [7]. Each application of PRO data is divided into four processes: patient identification (eg, registers, consecutive or individual referral), data collection (logistics and organization), data aggregation (at patient or at some specified group level) and the actual data use (Figure 2). In the present paper, the last two processes will be described together. The patient identification process involves the identification of patient(s) from whom data are to be collected, the data collection process involves the actual collection of health data, including logistic procedures, the data aggregation process involves the management and organization of collected data for the data use process, and the data use process involves the use of the health data for the purpose of the specified activity. Each process may be repeated or may take place simultaneously with the previous process (from [7]).

## Results

### Primary Findings

**Figure 3:**
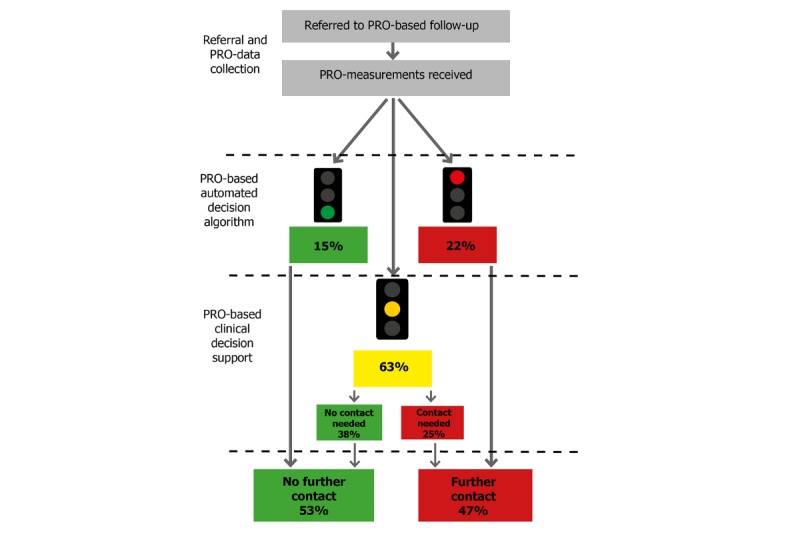
Flow chart for outpatients with epilepsy (AmbuFlex). Patients answer the disease-specific questionnaire at fixed intervals (eg, 3 months). In the first step, the answers are processed automatically based on a disease-specific algorithm. Green response: No need or wish for contact (a new questionnaire is scheduled in, eg, 3 months). Yellow response: May need contact (a clinician assesses the PRO overview (Figure 1) and other information to decide whether further contact is needed). Red response: Definite need or wish for contact (the patient is contacted). In total, only 47% of the patients are contacted in each round. PRO: patient-reported outcome.

An overview of selected projects is given in Table 2. In the first cohort studies, emphasis was on response rate, which was promoted by up to three reminders using email and letters and by giving the patient a free choice between Web and paper questionnaires. The maximum response rate was reached in patients with prostate cancer (Table 2), where the first questionnaire was answered by 95.9% of the patients. The PRO-based algorithm was first introduced in a screening program for patients with coronary heart diseases, and the AmbuFlex principle was fully unfolded in the implantation in epilepsy patients (Table 2, Figures 1 and 3).

**Figure 4:**
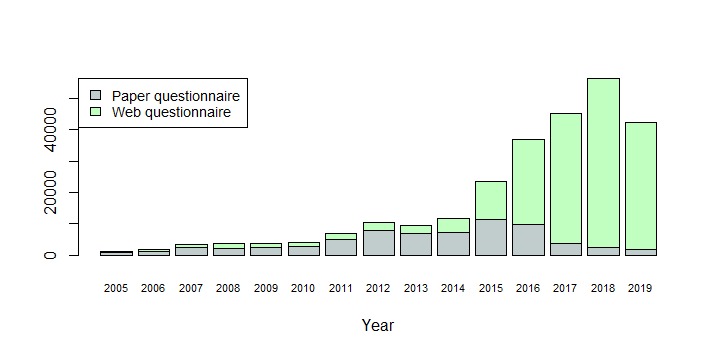
Questionnaires received by the AmbuFlex/WestChronic system 2005-19 by answering method.

**Figure 5:**
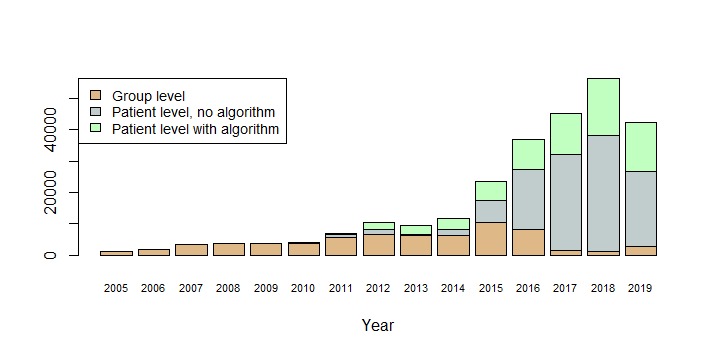
Questionnaires received by the AmbuFlex/WestChronic system 2005-19 by type of use.

Figure 4 and Table 3 show the development of the number of projects, patients, and received questionnaires. When considering that 2019 is only partly included, an exponential model of number of received questionnaires per calendar year explains 94% of the variation (Figure 4). At item level, a total of 13,538,760 responses have been received in the period. Until 5 years ago, web- and paper-based questionnaires were used with similar frequencies, but during the last 4 years web-based questionnaires have almost completely taken over. Projects where data was used at group level dominated until 2014, but PRO data used at the patient level is now by far the most frequent (Figure 5). Most projects and about one-third of the questionnaires now have an attached algorithm (AmbuFlex) by which the incoming answers are classified into two or three groups (Table 3 and Figure 3).

### Patient Identification

In the first part of the 15-year period, all projects were register-based, epidemiologic cohort studies with defined inclusion criteria, while from 2015 onwards PRO data for clinical use at the patient level has dominated the scene (Table 3 and Figure 5). In the latter case, patients are individually referred by a clinician. Patient cohorts are still created at group level and are used in research for topics like outcome measures, validation, and studies of determinants of referral. In 2019, 16,062 new patients were included, of which 73.8% (11,854/16,062) were referred individually by a clinician while the rest were included as part of a group by means of batch processing.

### Patient-Reported Outcome Data Collection

**Figure 6:**
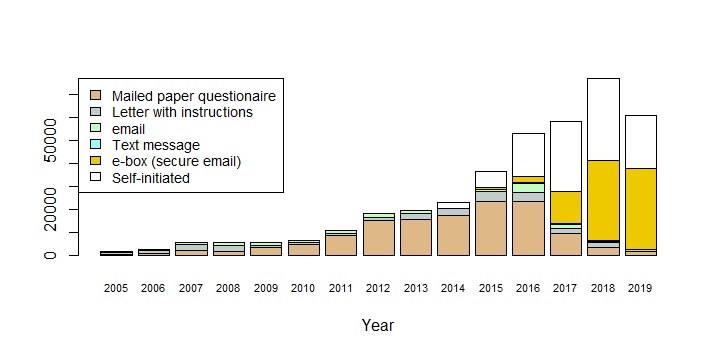
Contact to patients by year, and method for prompting the patients to fill in the questionnaire.

**Figure 7:**
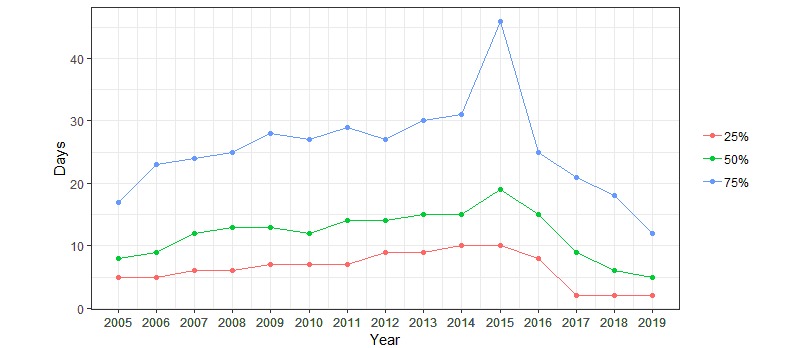
Turnaround time by year. Turnaround time is defined as the number of days from when the request is sent to the patient to when the answer is received.

Fundamental changes have taken place with respect to PRO data collection over the course of very few years. Until 2014, paper-based communication was used in more than 90% of the approaches (Figure 6), while electronic communication was predominantly used in epidemiological studies with multiple measurements where about half of the communication was electronic (emailing and web-based answering) (Figures 4 and 6). A minor revolution took place November 1, 2014. From this date, all communication between the Danish authorities (including all public hospitals), and Danish citizens had to use a national, secure electronic mail system, called e-boks. All citizens are provided an e-boks, but while its use was previously voluntary, only people with disabilities can now be granted a dispensation and continue to receive paper letters from places like hospitals. After the AmbuFlex/WestChronic system gained access to e-boks (Table 1), this channel rapidly became the most important communication method to reach patients (Figure 6). These changes in communication methods to and from patients had consequences for turnaround time (ie, the time from asking the patient to answer until the questionnaire is received) (Figure 7). A total of 75% of the answers were received within 17 days in 2005 due to the homogeneous cohorts, but turnaround time increased slowly until 2014. At this time, the Danish Mail decreased their services due to economic problems caused by the steep fall in paper-based mail. The historic low turnaround time in 2019 is caused by a combination of e-boks communication and the web-answering method. Today, the first answers are received within minutes. Use of ordinary, unsecure email ended in 2018. Text messaging is still used for reminders in some projects but is mainly used as a 2-factor authentication tool, where the patient, after entering their credentials (CPR number), receives a 6-digit cipher code as a text message.

**Table 2:** Characteristics of selected PRO-based projects implemented in the AmbuFlex/WestChronic system.

Studies		Characteristics	Description
**Group-level projects**				
	C50 Breast cancer	PRO^a^ data collection: 2004-14 107 items/questionnaire 1008 patients (100% females) 4731 questionnaires (43.1% web) Follow-up^b^: 4.7 (9.1) years Papers: 3 [8-10]	The source population was women referred to mammography at two regional hospitals in Region Central Denmark on clinical suspicion of breast cancer. The women were mailed a baseline questionnaire which was filled in and returned before the date of mammography. All respondents were subsequently interviewed by telephone and invited to join the follow-up study, irrespective of diagnosis. Women diagnosed with breast cancer (n=256; 7.2% of respondents) as well as a sample of women without cancer (n=291) were followed every 3 months for up to 9 years with questionnaires including generic scales on fatigue (MFI-20^c^) [11], depression and anxiety (HADS^d^) [12], and selected items from SF-36^e^ [13]. Inclusion in the study ended when a national plan for fast-track diagnosing of cancer was implemented in 2008, with which the design was incompatible since baseline data could no longer be collected before diagnosis. The final cohort size was 60% of the planned size. Register information on treatment and survival were collected from national cancer databases.
	I20 Ischemic heart diseases	PRO data collection: 2006-13 103 items/questionnaire (1,323 patients (20.4% females) 13,171 questionnaires (40.3% web) Follow-up: 3.4 (7.0) years Papers: 6 [14-19]	Patients treated with percutaneous coronary intervention at Aarhus University Hospital, which at that time performed this procedure on behalf of the entire Region Central Denmark (1.3 million inhabitants). Patients <80 years old were included based on records in the hospital administration system. Patients were followed up with questionnaires every fourth month for up to 7 years, including HADS [12], MFI-20 [11], IPAQ^f^ [20], selected items from SF-36 [13], items related to the psychosocial job-strain model [21], the Seattle angina questionnaire [22], and questions on lifestyle and rehabilitation. Register information on treatment, survival, and sick leave was collected from national databases.
	I64 Stroke	PRO data collection: 2009-14 21 items/questionnaire 2618 patients (39.1% females) 9622 questionnaires (25.3% web) Follow-up: 2.1 (5.1) years Papers: 2 [23,24]	Patients with first-time stroke in Region Central Denmark were included prospectively based on online access to a national, disease-specific register. Patients were followed with questionnaires every fourth month for up to 5 years, including HADS [12], MFI-20 [11], WHODAS^g^ [25], and selected items from SF-36 [13]. Register information on treatment, survival, and sick leave was collected from national databases.
	C61 Prostate cancer	PRO data collection: 2011-17 32 items/questionnaire 13,434 patients (0.0% females) 16,066 questionnaires (15.1% web) Follow-up: 1.7 (3) years	The Danish quality database DaProCa has recorded clinical information nationwide in patients with prostatic cancer since 2010 [26]. In 2011, it was decided that PRO information should also be included to better describe the treatment outcome. Patients were identified in the register and mailed questionnaires 1 year and 3 years after initial diagnosis. The project was the first where an initial response rate above 90% was obtained. The PRO data in the quality databases have, however, only been used sporadically.
**Patient-level projects**				
	I20 Ischemic heart diseases	PRO data collection: 2011-17 14 items/questionnaire 5097 patients (40.5% females) 5121 questionnaires (19.4% web) Papers: 1 [27]	This project was the first to use automatic algorithm-based handling of questionnaires. Patients admitted to Hospital Unit West Jutland for treatment of ischemic heart disease were identified based on ICD-10^h^ diagnoses in the business intelligence register in Region Central Denmark. Patients were mailed a questionnaire containing the HADS [12]. An algorithm embedded in the AmbuFlex/WestChronic system processed the incoming answers and printed individualised letters with the results. Patients were advised to contact their GP^i^ if depression or anxiety scores were above the established thresholds, which was the case in 30.2% of the responses. An analysis based on register information on consultations with a GP or psychologist revealed that only a few additional cases of depression were detected.
	G40 Epilepsy	6 departments PRO data collection: 2012-present 47 items/questionnaire 6405 patients (50.5% females) 21,296 questionnaires (56.7% web) Follow-up: 3.4 (7.6) years Papers: 6 [5,28-32]	This project was the first project where PRO data were used as the basis for outpatient follow-up (AmbuFlex) and was developed in close cooperation with the Department of Neurology, Aarhus University Hospital. Patients with epilepsy attending neurological outpatient clinics are individually referred to AmbuFlex follow-up. Instead of fixed appointments at the clinic every 3, 6, or 12 months, the patients are prompted to answer a short disease-specific questionnaire developed in cooperation with the clinicians. Based on an automated algorithm, red and yellow flags as well as patient preferences are identified. Patients with flags or a wish for contact are included on the clinicians online to-do list, and the PRO overview is displayed to the clinicians within the Electronic Health Record system (Figure 1). Questionnaires with no flags and no wish for a contact are handled automatically by AmbuFlex and a new questionnaire (eg, 3 months later), is scheduled. Overall, 53% of the PRO-based contacts are handled without further contact to the patient (Figure 3) This was the first of three AmbuFlex projects implemented on a national basis 2013 [33].
	C34 Lung cancer	8 departments PRO data collection: 2014-present 52 items/questionnaire 2274 patients (50.1% females) 12,658 questionnaires (100% web) Follow-up: 0.6 (4.6) years	Patients treated for lung cancer were asked by the front-desk staff to fill in the online questionnaire in the waiting area at each follow-up outpatient clinic visit. The intention was to use the PRO information in the consultation a few minutes later. The project was implemented at seven departments throughout Denmark in cooperation with the Danish Cancer Society. Log-files in the AmbuFlex/WestChronic system are kept to document each time patient data are displayed and to whom. On average, only 47% of the questionnaires were viewed by a clinician (Figure 7), with huge differences between hospitals ranging from 14-93% [34].
	M05 Rheumatoid arthritis	2 departments PRO data collection: 2014-present 40 items/questionnaire 676 patients (69.7% females) 2785 questionnaires (84.5% web) Follow-up: 1.7 (5.0) years Publications: 1 [35]	The project started as a non-inferiority randomized controlled trial conducted in cooperation with Rheumatologic Department, Aarhus University Hospital, where patients were randomized to PRO-based telehealth or conventional outpatient follow-up. Disease activity was measured by the Danish version of the Flare-RA instrument [36], and the primary outcome was a change in the DAS28^j^. PRO-based tele-health achieved disease control like that of conventional outpatient follow-up. All patients were contacted. The project has continued as an AmbuFlex project with a similar questionnaire, where only patient answers with red or yellow flags are assessed and patients contacted if needed.
	C80 Side effects during antineoplastic treatment	2 departments PRO data collection: 2015-present 60 items/questionnaire 7011 patients (57.8% females) 59,202 questionnaires (100% web) Follow-up: 0.3 (4.2) years	In cancer treatment, questions on toxicity symptoms are normally not asked and registered systematically, and the ongoing therapy is therefore not evaluated in accordance with the present state of the patient. In several AmbuFlex projects, PRO-based self-reports are used during the period the patient is receiving chemotherapy in an outpatient setting. PRO data are used to decide if the planned chemotherapy should be postponed or adjusted. The PRO measures used include items from PRO-CTCAE^k^ [37], EORTC^l^ [38] and PRO measures based on ad hoc developed single red-flag items.
	C34 Lung cancer	6 departments PRO data collection: 2018-present 17 items/questionnaire 69 patients (56.7% females) 973 questionnaires (100% web) Follow-up: 0.2 (0.9) years	PRO-based systematic symptom monitoring may improve overall survival in cancer patients who are followed up with after their initial treatment [39,40]. In this Danish multicenter RCT study, we compare standard follow-up with weekly PRO measurements where red flag responses are automatically reported to clinicians for further evaluation.
	C80 Cancer, inpatients	1 department PRO data collection: 2017-present 22 items/questionnaire 868 patients (60.9% females) 4849 questionnaires (100% web) Follow-up: 0.1 (1.8) years	Clinical use of PRO measures often includes only outpatients. In this developmental implementation of AmbuFlex, PRO data are used in inpatients to support the dialogue between the patient, the nurse, and the doctor while the patient is hospitalized, and they are used to prioritize patients to be discussed during the daily rounds.
	G40 Epilepsy	2 departments PRO data collection: 2015-present 34 items/questionnaire 182 patients (45.6% females) 449 questionnaires (31.9% web) Follow-up: 2.2 (4.3) years	In all patient groups, a proportion of patients are not capable of answering a questionnaire. Some patients suffering from epilepsy live in institutions or are taken care of by their next of kin. These patients may be at increased risk of having important symptoms left unnoticed by the health care system in connection with normal follow-up, since the person accompanying the patient to the hospital may not be the person who has the most knowledge about the patient. In AmbuFlex/Epilepsy, a specific proxy questionnaire was developed with an algorithm like that used by the other epilepsy patients.

^a^PRO: patient-reported outcome.

^b^Median follow-up with maximum in parenthesis. Based on patients who have answered at least two questionnaires by September 8, 2019.

^c^MFI: Multidimensional fatigue inventory.

^d^HADS: Hospital Anxiety and Depression Scale.

^e^SF: Short Form Health Survey.

^f^IPAQ: International Physical Activity Questionnaire.

^g^WHODAS: World Health Organization disability assessment schedule 2.0.

^h^ICD-10: 10th edition of the International Statistical Classification of Diseases and Related Health Problems.

^i^GP: general practitioner.

^j^DAS: disease activity score.

^k^PRO-CTCAE: patient-reported outcome measure–Common Terminology Criteria for Adverse Events.

^l^EORTC: European Organization for Research and Treatment of Cancer.

**Table 3:** Summary of PRO-based projects by year from 2005-2019.

Year	Projects	ICD-10^a^ groups	Level of aggregation, %			Departments^b^	Patients^b^	Questionnaires^b^
			Group	Patient, no algorithm	Patient, with algorithm			
2005	1	1	100	0	0	0	542	1200
2006	2	2	100	0	0	0	923	1876
2007	2	2	100	0	0	0	1318	3421
2008	2	2	100	0	0	0	1232	3630
2009	3	4	80	20	0	1	1606	3747
2010	2	4	75	25	0	1	2214	4098
2011	6	6	57	14	29	13	4721	6848
2012	8	7	67	0	33	17	7733	10,434
2013	11	10	58	0	42	10	7174	9346
2014	16	11	35	12	53	23	8415	11,816
2015	17	14	17	22	61	39	16,490	23,444
2016	23	16	13	25	63	49	20,201	36,912
2017	34	20	18	21	62	67	18,721	45,058
2018	44	24	14	27	59	91	21,143	56,178
2019^c^	47	26	15	27	58	97	18,262	42,127
Total	64	28	28	26	46	141	78,980	260,433

^a^ICD-10: 10th edition of the International Statistical Classification of Diseases and Related Health Problems.

^b^Patients and departments may be involved in more than one disease-specific project.

^c^As of September 8, 2019.

### Patient-Reported Outcome Data Aggregation and Data Use

**Figure 8:**
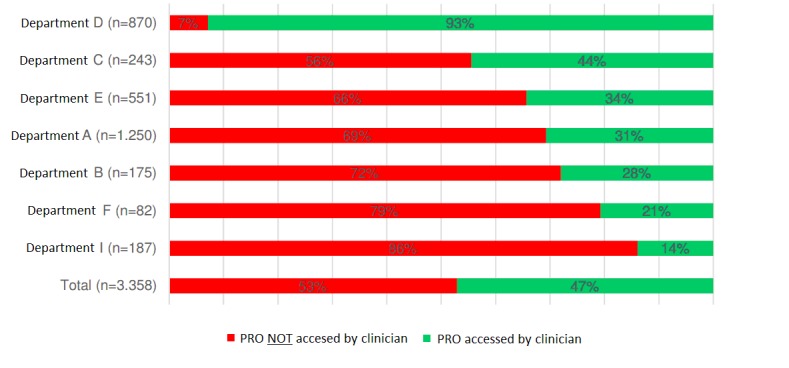
The proportion of PRO questionnaires that was viewed by a clinician during the follow-up visit of patients treated for lung cancer in seven Danish oncological departments (translated from Danish [34]). PRO: patient-reported outcome.

While data from group-level projects are aggregated and used in the analysis phases of each epidemiological study, data in patient-level projects are, or should be, aggregated and used the date the questionnaire is received; however, this is not always the case. If PRO data are used as an add-on in connection with ordinary organized outpatient follow-up, it is up to each clinician to decide to open the PRO overview or not. In a multicenter project covering eight Danish hospitals, the patients treated for lung cancer answered a PRO questionnaire each time they went to the hospital for a follow-up visit, with the idea being that the PRO data should be used to enforce patient-clinician communication during the consultation (Table 2). For legal reasons, the AmbuFlex/WestChronic system keep automated log files of when and to whom the PRO data are shown. In total, a minority of responses (47%) were seen by a clinician (Figure 8). There were major differences between departments, ranging from 14-93% [34]. Similar figures have been found in other projects where the PRO data are an add-on to the existing outpatient set-up. In AmbuFlex, however, the PRO data are not an add-on but the basis for the follow-up. Each time a questionnaire is received, it is either handled automatically (green response, Figure 3) or included on a list where it remains until a clinician has accessed it and made a clinical decision regarding whether the patient should be contacted or not. Therefore, virtually no AmbuFlex questionnaires remain unnoticed.

## Discussion

During the 15-year period, we have experienced an exponential growth in the number of answered questionnaires and several marked changes have taken place. The creation of cohorts for clinical epidemiological research purposes has shifted towards cohorts defined by clinical use of PRO data at the patient level, and PRO-based follow-up (AmbuFlex) has replaced fixed appointments in many patient groups. Overall, the system is currently in use in 47 International Statistical Classification of Diseases and Related Health Problems (ICD)–groups, covering 16,000 patients and 94 departments throughout Denmark. In the first part of the period, a combination of paper- and web-based questionnaires secured response rates above 90% and low attrition rates. Back in 2005 66.5% questionnaires were paper based, but this is only the case for 4.3% in 2019. In AmbuFlex even higher response-rates have been reached, but the numbers are not directly comparable because each patient is individually referred and has given consent to participate. In addition, the approach methods for questionnaires and reminders have changed from letters, emails, and text messaging to a national, secure, electronic mail system through which 93.2% of communication to patients took place in 2019. The combination of secure email and web-based answering has resulted in a low turnaround time, where half of all responses are received within 5 days.

Our experiences probably reflect general changes that have taken place during the 15-year period. First, the demand for PRO measurements has increased, quantitatively as well as qualitatively. PRO measures have been used at the group level for many years, even before the PRO term was coined, when it was termed Quality of Life measures among other things (particularly in clinical trials and observational studies). PRO measures have also been used at the group level to provide evidence for drug and device approval and, in some countries, used to evaluate quality of care and health service provider performance. By contrast, using PRO data systematically at the individual patient level might be relatively new, but it is now by far our predominant activity. This increased demand is driven by a wish among patients, as well as clinicians, to use the potential in PRO data to promote better symptom assessment, more patient-centered care, and more efficient use of resources. In Denmark, hospital administrators and health ministerial civil servants quickly realized PRO measures’ potential to prioritize resources to the outpatients who actually want or need clinical attention.

Second, substantial technological changes have occurred in the period, which has created new opportunities. For example, an electronic health record (EHR) system has been implemented that covers the whole Region Central Denmark, and AmbuFlex obtained an early online connection that allowed clinicians to access it because it was a part of the EHR. In addition, the national secure electronic mail system (e-boks) has had a high impact on reducing response time.

Finally, several legal changes have occurred. The rationale for implementation of the General Data Protection Regulation (GDPR) is sound because there is a real risk of patients’ information being accessed and used by people for whom it was not initially intended. However, much time is used on details and bureaucratic documentation procedures, which doubly enhance data security. Another legal change is the European Union’s medical device regulation. Questionnaires with an attached algorithm, like those used in AmbuFlex, are classified as a medical device and as such they must be certified with a certification (Conformité Européenne) marking. Patient safety is a cornerstone, but since we are dealing with outpatients who are instructed to contact their family doctor or the department directly in case of exacerbation it makes little sense to treat a questionnaire with the same rules as electronic medical equipment. At present, AmbuFlex is granted a dispensation for current projects until the marking is in place, but not allowed to launch new projects. If AmbuFlex were to start today, it is unlikely that we would ever have surfaced.

One issue, which is often overlooked, is whether collected PRO data are used sufficiently [7,41]. Most patients are very careful when filling in their questionnaires, and it should be our obligation to promote as much use of the data as possible, irrespective of if data are originally collected for research, quality assessment, or clinical use at the patient level. In the future, clinical settings will probably be the most important source of PRO data, but also for other purposes like research and quality surveillance, so new ways to conduct complementary data collection will be necessary [7]. The current legal restrictions on the joint use of health data imposed by the GDPR make no distinction between use and misuse, and steps should be taken to alleviate these legal restrictions on the joint use of PRO data.

## Abbreviations

CPR: Civil Personal Registration
DAS: disease activity score
EHR: electronic health record
EORTC: European Organization for Research and Treatment of Cancer
GDPR: General Data Protection Regulation
GP: general practicioner
HADS: Hospital Anxiety and Depression Scale
ICD: International Statistical Classification of Diseases and Related Health Problems
IPAQ: International Physical Activity Questionnaire
MFI: multidimensional fatigue inventory
PRO: patient-reported outcome
PRO-CTCAE: patient-reported outcome measure–Common Terminology Criteria for Adverse Events
SF: Short Form Health Survey
WHODAS: World Health Organization disability assessment schedule 2.0
